# Complete Genome Sequence of a Highly Pathogenic Avian Influenza Virus (H5N2) Associated with an Outbreak in Commercial Chickens, Iowa, USA, 2015

**DOI:** 10.1128/genomeA.00613-15

**Published:** 2015-06-11

**Authors:** Travis Clement, Gerald F. Kutish, Jill Nezworski, Joy Scaria, Eric Nelson, Jane Christopher-Hennings, Diego G. Diel

**Affiliations:** a Animal Disease Research and Diagnostic Laboratory (ADRDL), Department of Veterinary and Biomedical Sciences, South Dakota State University, Brookings, South Dakota, USA; b Department of Pathobiology, University of Connecticut, Storrs, Connecticut, USA; c Blue House Veterinary LLC, Hector, Minnesota, USA

## Abstract

A novel reassortant influenza A virus (H5N2) was first detected in British Columbia, Canada, in December 2014. The virus rapidly spread along the waterfowl migration flyways in the United States, causing multiple HPAI outbreaks in poultry. Here, we present the complete genome sequence of HPAIV-H5N2 from a commercial chicken flock in Iowa.

## GENOME ANNOUNCEMENT

A novel reassortant highly pathogenic avian influenza (HPAI) H5N2 virus containing gene segments related to Eurasian H5N8 and to North American lineage waterfowl viruses was first detected in North America in December 2014 in British Columbia, Canada (1). The virus was initially isolated in the United States on 8 December 2014 from a northern pintail (*Anas acuta*), which was found dead in Whatcom County, Washington (2). Since then, related viruses have been associated with high-mortality avian influenza (AI) outbreaks in backyard and commercial U.S. poultry flocks along the Pacific, Central, and Mississippi waterfowl migration flyways (3, 4). Here, we report the complete genome sequence of an HPAI H5N2 virus associated with a high-mortality AI outbreak in a commercial layer flock located in the State of Iowa in the Mississippi flyway.

On 19 April 2015, a sudden increase in mortality (over 30,000 birds) was observed in a large commercial chicken flock located in the state of Iowa. Oropharyngeal swab samples were collected from affected chickens and submitted to the South Dakota Animal Disease Research and Diagnostic Laboratory (ADRDL) for diagnostic investigation. Real-time reverse transcription PCR performed on RNA samples extracted from oropharyngeal swabs were positive on the standard National Animal Health Laboratory Network (NAHLN) influenza matrix PCR and the H5 subtype AIV assays, whereas all samples were negative on the H7 subtype AIV PCR test. Further testing of the positive samples at the National Veterinary Services Laboratory confirmed the presence of the Eurasian H5 clade 2.3.4.4 virus, and partial sequence of hemagglutinin (HA) and neuraminidase (NA) revealed 99% nucleotide identity with the U.S. H5N2 index case A/Northern pintail/Washington/40964/2014 (2).

Using a universal influenza A amplification kit (PathAmp FluA reagents; Life Technologies) followed by next-generation sequencing at the SD ADRDL (Nextera XT DNA library preparation kit and MiSeq reagent nano kit v2; Illumina), the full sequences of the eight influenza A genome segments were obtained directly from RNA extracted from pooled diagnostic specimens from the outbreak. Genome segments were assembled using the CAP3 assembly server (5), and BLAST searches were conducted to determine the nucleotide identity of the genes of the influenza A/chicken/Iowa/04-20/2015 virus. Results from the BLAST search revealed that all genome segments of A/chicken/Iowa/04-20/2015 share 99% nucleotide identity with the U.S. H5N2 index case A/Northern pintail/Washington/40964/2014 (2) (GenBank accession numbers KP307973 to KP307980). BLAST results of the HA gene segment revealed 100% nucleotide identity with influenza A virus isolate A/turkey/Washington/61-22/2014(H5N2) (GenBank accession number KP739397) associated with the initial outbreaks of HPAI in poultry in Washington. The rapid spread of this novel reassortant H5N2 HPAI virus, its continuous circulation in wild birds, and frequent infections of commercial poultry flocks (4) pose significant challenges to control the ongoing HPAI outbreak in the United States.

### Nucleotide sequence accession numbers.

The genome sequence of A/chicken/Iowa/04-20/2015(H5N2) virus is available in GenBank under the accession numbers KR492971 to KR492978.

## References

[1] Pasick J, Berhane Y, Joseph T, Bowes V, Hisanaga T, Handel K, Alexandersen S 2015 Reassortant highly pathogenic influenza A H5N2 virus containing gene segments related to Eurasian H5N8 in British Columbia, Canada, 2014. Sci Rep 5:9484. doi:10.1038/srep09484. 25804829 PMC4372658

[2] Ip HS, Torchetti MK, Crespo R, Kohrs P, DeBruyn P, Mansfield KG, Baszler T, Badcoe L, Bodenstein B, Shearn-Bochsler V, Killian ML, Pedersen JC, Hines N, Gidlewski T, DeLiberto T, Sleeman JM 2015 Novel Eurasian highly pathogenic avian influenza A h5 viruses in wild birds, Washington, USA, 2014. Emerg Infect Dis 21:886–890. doi:10.3201/eid2105.142020. 25898265 PMC4412248

[3] Jhung MA, Nelson DI, Centers for Disease Control and Prevention (CDC) 2015 Outbreaks of avian influenza A (H5N2), (H5N8), and (H5N1) among birds—United States, December 2014-January 2015. MMWR Morb Mortal Wkly Rep 64:111. 25654614 PMC4584850

[4] APHIS/USDA 2015 Avian influenza disease. http://www.aphis.usda.gov/wps/portal/aphis/ourfocus/animalhealth/sa_animal_disease_information/sa_avian_health/ct_avian_influenza_disease.

[5] Huang X, Madan A 1999 CAP3: a DNA sequence assembly program. Genome Res 9:868–877. doi:10.1101/gr.9.9.868. 10508846 PMC310812

